# What Works and What Doesn’t Work? A Systematic Review of Digital Mental Health Interventions for Depression and Anxiety in Young People

**DOI:** 10.3389/fpsyt.2019.00759

**Published:** 2019-11-13

**Authors:** Sandra Garrido, Chris Millington, Daniel Cheers, Katherine Boydell, Emery Schubert, Tanya Meade, Quang Vinh Nguyen

**Affiliations:** ^1^MARCS Institute for Brain, Behaviour & Development, Western Sydney University, Milperra, NSW, Australia; ^2^Translational Health Institute, Western Sydney University, Campbelltown, NSW, Australia; ^3^School of Social Sciences & Psychology, Western Sydney University, Milperra, NSW, Australia; ^4^NSW Health, Sydney, NSW, Australia; ^5^Blackdog Institute, Randwick, NSW, Australia; ^6^School of Arts & Humanities, University of New South Wales, Kensington, NSW, Australia; ^7^School of Computing & Engineering, Western Sydney University, Parramatta, NSW, Australia

**Keywords:** children, adolescents, unguided self-help, self-management, low mood, prevention

## Abstract

**Background:** A major challenge in providing mental health interventions for young people is making such interventions accessible and appealing to those most in need. Online and app-based forms of therapy for mental health are burgeoning. It is therefore crucial to identify features that are most effective and engaging for young users.

**Objectives:** This study reports a systematic review and meta-analysis of digital mental health interventions and their effectiveness in addressing anxiety and depression in young people to determine factors that relate to outcomes, adherence, and engagement with such interventions.

**Methods:** A mixed methods approach was taken, including a meta-analysis of 9 randomized controlled trials that compared use of a digital intervention for depression in young people to a no-intervention control group, and 6 comparing the intervention to an active control condition. A thematic analysis and narrative synthesis of 41 studies was also performed.

**Results:** The pooled effect size of digital mental health interventions on depression in comparison to a no-intervention control was small (Cohen’s d = 0.33, 95% CI 0.11 to 0.55), while the pooled effect size of studies comparing an intervention group to an active control showed no significant differences (Cohen’s d = 0.14, 95% CI -.04 to 0.31). Pooled effect sizes were higher when supervision was involved (studies with no-intervention controls: Cohen’s d = 0.52, 95% CI 0.23 to 0.80; studies with active control: Cohen’s d = 0.49, 95% CI -0.11, 1.01). Engagement and adherence rates were low. Qualitative analysis revealed that users liked interventions with a game-like feel and relatable, interactive content. Educational materials were perceived as boring, and users were put off by non-appealing interfaces and technical glitches.

**Conclusions:** Digital interventions work better than no intervention to improve depression in young people when results of different studies are pooled together. However, these interventions may only be of clinical significance when use is highly supervised. Digital interventions do not work better than active alternatives regardless of the level of support. Future interventions need to move beyond the use of digital educational materials, considering other ways to attract and engage young people and to ensure relevance and appeal.

## Introduction

In Australia, approximately 8% of young people between 11–17 years of age meet the DSM criteria for major depressive disorder (MDD), while about 20% report high levels of psychological distress (1). The rates of MDD may be as high as 11% in youths in the U.S. (2). In fact, suicide is the second leading cause of death among 15–29-year-olds globally (3). In addition, depression is highly under-diagnosed and thousands who fall outside these statistics experience its debilitating effects on functioning at an important developmental stage. Depression and other mental illnesses affect the social and intellectual development of young people, reducing engagement with education, and if untreated, can become lifelong disabilities (4).

Despite the importance of addressing mental illness early only 20–40% of youths in need in Australia (1) and 25% of youths in the U.K. receive professional help (5). This low engagement with mental health services appears to occur for a variety of reasons: the lack of motivation inherent in conditions such as depression (6), low rates of mental health literacy (7), and the stigma, discrimination and embarrassment surrounding mental illness (8). Young people are also still developing skills in executive functioning such as self-monitoring and organization, which are necessary to identify a mental health problem and obtain support (9).

Although few young people seek professional help, during episodes of depression consumption of media such as music, internet, and television increases (10). Thus Digital Mental Health Interventions (DMHIs) are increasingly of interest as a solution to the low help-seeking and uptake rates of professional mental health services. Studies tend to support the effectiveness of self-help mental health programs whether digital or otherwise, which can be as effective if not more effective than face-to-face delivery (11). Numerous studies have demonstrated the usefulness of web-based programs (12, 13). Young people report feeling more comfortable discussing sensitive and personal issues in the relative anonymity of an online context and use the internet as a major source of mental health information (14, 15). Mobile “apps” are proving particularly useful for administering DMHIs because of the widespread ownership of mobile phones, with the majority of young people in the U.S. reporting almost constant usage of smartphones (16). Several reviews of smartphone applications for mental health across age groups have reported positive benefits (13, 17).

Notably, however, many apps for depression and anxiety that are currently available are not evidence-based and may thus actually be harmful to people with mental illness (18). Even among those purporting to be drawn from evidence-based therapies such as cognitive behavioural therapy, only a small percentage actually contain the core principles of those therapeutic traditions (19). Furthermore, Hollis and colleagues (20) in their meta-review reported that while there is some evidence in support of the effectiveness of DMHIs for depression and anxiety in young people, studies are methodologically limited making it difficult to draw clear conclusions. Furthermore, they suggest the need for identification of the components that make DMHIs effective such as human interaction.

In fact, human interaction has been identified as an important factor influencing effectiveness and engagement with DMHIs (21), but it may also detract from the cost-effectiveness of DMHIs in comparison to face-to-face treatment (20). Furthermore, other disadvantages to the inclusion of social features exist, such as unhelpful advice from peers, and the possibility that some youths may feel afraid to share personal problems even anonymously.

The aim of the current review, therefore, is to examine the literature about DMHIs to address mental health in young people. We have focused on depression and anxiety as these are among the most prevalent mental health conditions experienced by young people and often co-occur with many other disorders (22–24). Specifically we aimed to investigate:

1. Do DMHIs reduce anxiety and depression in young people aged 12–25 compared to no intervention or an active control group?
2. How effective are DMHIs in reducing anxiety and depression in young people when interaction with the intervention is unsupervised?
3. What features and components of DMHIs are most liked or disliked by young users?

## Method

### Study Design

Given the focus in the current review on both effectiveness and engagement, it was expected that the literature reviewed could include both quantitative and qualitative data. Therefore, a mixed methods approach was selected. Mixed methods reviews attempt to combine looking at ‘what works’ with ‘how and why it works’, combining varied research methods in the analysis or in the types of studies reviewed (25). Mixed method reviews can provide a more holistic understanding than a meta-analysis alone since they are able to integrate a wider variety of studies and provide insights into mechanisms and processes.

### Identification and Selection of Studies

A systematic search was conducted in PsychInfo, PubMed, Proquest, and Web of Science using the following terms in the title, abstract and subject descriptors: “mobile”, “application”, “smartphone”, “mobile phone”, “cell phone”, “text message”, “internet-administered therapy”, “computer-aided therapy”, “online” AND “depression”, “anxiety” AND “youth”, “young person”, “adolescent”. The initial search returned 4,828 articles. With duplicates deleted this number was reduced to 3,352.

Identified references were screened according to the following inclusion criteria: (1) participants aged 12 to 25, (ii) interventions targeting depression or anxiety, (iii) interventions delivered by computer, on smartphones, or online, (iv) studies published between 2007 and 2017. We also excluded reviews, opinion, or discussion pieces and unpublished works. While quality assessment was part of the review process, studies were not excluded on the basis of study type or quality since the mixed methods approach taken allowed the inclusion of varied methodologies.

**Figure 1:**
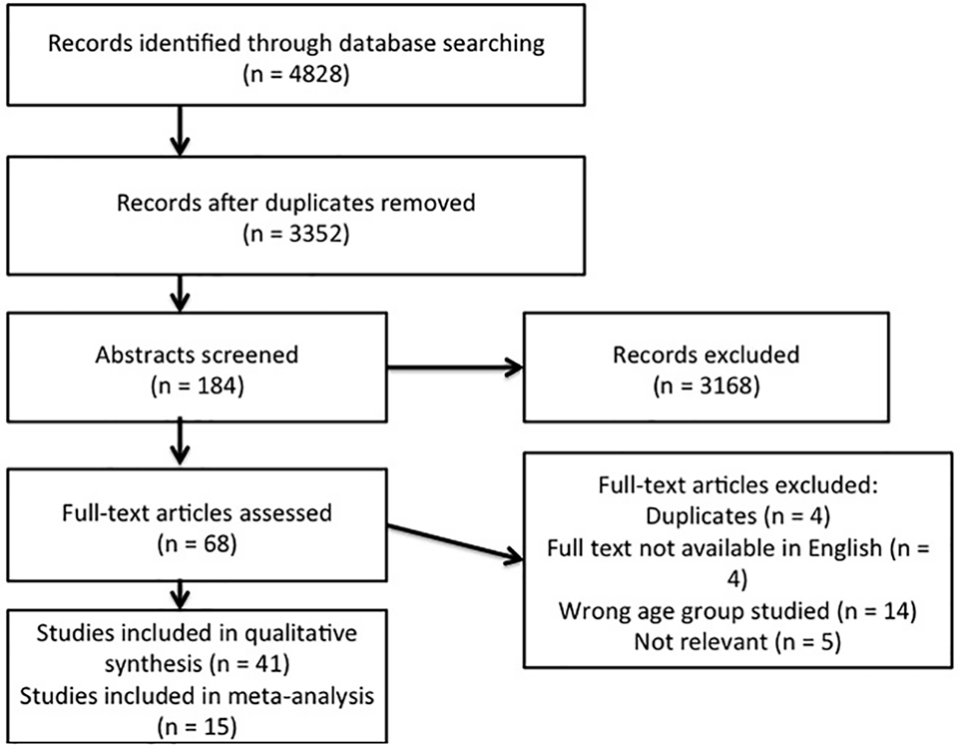
PRISMA flowchart of article selection.

After title screening by 1 researcher 184 abstracts were uploaded to Covidence, an online platform for conducting systematic reviews (http://www.covidence.org). These were scrutinised by 2 researchers. Once agreement was reached on eligibility, 68 articles remained. Full text appraisals were then conducted by 2 researchers and a further 27 articles not meeting the inclusion criteria were excluded, leaving 41 in the review (**Figure 1** and **Supplementary Materials**).

### Quality Assessment and Data Extraction

Two researchers independently conducted the data extraction. Information about the characteristics of the studies, participants, interventions, and final outcomes were entered into a pre-established template in Covidence. Qualitative data from the studies relating to usability and appeal of the interventions was also extracted. The quality of studies was assessed using the Joanna Briggs Institute’s (26) critical appraisal tools and the CONSORT-EHEALTH Checklist (V.1.6.1) (27). Studies were considered methodologically sound if they had a matched control group, pre-post data, and randomization, and were only included in the meta-analyses if they met these criteria. However, since this is a mixed-methods review, studies not meeting the criteria for inclusion in the meta-analysis were retained in the study to form part of the narrative synthesis. Risk of bias was assessed using the standard Cochrane Risk of Bias tool (28) in Covidence, which poses questions about aspects of trial design, conduct, and reporting which the user rates as ‘Low’ or ‘High’ risk of bias for each study. Single cohort studies and RCTs were assessed using different indicators of bias under the 5 categories of possible bias as outlined in the *Cochrane Handbook of Systematic Reviews* (29), and a overall assessment of bias as ‘Low’ or ‘High’ given for each study. Where not enough details were reported in the study to assess risk of bias, this was labeled ‘Unclear’. Again, however, studies with some risk of bias were not excluded from the analyses but sub-group comparisons were conducted to assess whether results differed depending on bias.

### Analysis

#### Meta-Analysis

Due to a lack of comparable randomized controlled trials (RCTs) for anxiety, the meta-analysis focused on DMHIs targeting depression. For each RCT of a DMHI for depression the effect size indicating the difference between the intervention group and the control group at post-test was calculated in OpenMeta Analyst (30). Effect size calculations (Cohen’s *d* or standardized mean differences) were conducted using the means and standard deviations of post-test scores on instruments measuring symptoms of depression such as the depression subscale of the Depression, Anxiety, Stress, Scale (DASS, 31) or the Centre for Epidemiological Studies Depression Scale (CES-D, 32). Scores on these measures immediately after the study were used rather than longitudinal follow-up scores due to a lack of consistent reporting across the studies. Sample sizes used were the number of participants with complete data in each group rather than intention-to-treat numbers. If effect sizes could not be calculated due to a lack of information, the study was excluded from the effectiveness analysis. Pooled mean effect sizes were calculated using the random effects model as considerable heterogeneity between studies was expected. These were calculated separately for studies using a no-intervention control group and those using an active control group. Subgroup analyses were conducted to investigate the impact of other variables on effectiveness by testing for significant differences between subgroups using a fixed effects model. In particular a sub-group analysis according to risk of bias was conducted to assess how study design contributed to outcomes. Since one area of interest in this review was to investigate whether DMHIs are useful for the high percentages of young people not obtaining professional help, we also conducted a sub-group analysis according to the level of supervision or interaction involved in the treatment condition.

#### Narrative Synthesis

All studies including those not in the meta-analysis were evaluated using a narrative synthesis model (33). Qualitative data included both results of interviews with participants, and descriptions of survey results relating to the appeal of DMHIs. For example, some studies reported direct comments from participants about their experiences using the DMHI, while others reported levels of agreement with statements about appeal and quality. These data were coded by 2 researchers using standard techniques for thematic analysis to generate an understanding of features that are appealing and aspects that promoted engagement and adherence (34). Codes were grouped to detect patterns, and themes were identified and defined. Once consensus was reached the lead author prepared a narrative analysis, which was checked independently by the other authors.

## Results

### Study and Participant Characteristics

The 41 studies included 11 from Australia, 10 from the U.S., 12 from other English speaking countries, 5 from Northern Europe, 2 from Asia, and one from South America. The majority of studies were RCTs (n = 27) (**Table 1**), while 13 were single cohort studies (including 4 with pre-post designs), and one used a case study methodology. Most of the studies used participants recruited from educational institutions (n = 20), 9 were conducted in mental health care settings, 4 with the general community, 4 in primary care settings, 2 in hospitals, and one in a youth organisation. One study did not report recruitment methods or participant information in enough detail to determine the setting.

**Table 1:** Study and Participant Characteristics.

Paper	Study Design	Recruitment Setting	Population	Severity at baseline	Total Sample	Age Mean (SD)	Age Range	Female %
Anstiss and Davies (35)	Single cohort	Youth helpline	Depression or anxiety	Mild-mod	21	19.3 (2.8)	12–24	66.7
Bobier et al. (36)	Single cohort	Mental health facility	Mental illness of any type	Severe	20	16.5 (0.7)	Not reported	40
Bradley et al. (37)	Single cohort (pre-post design)	Children’s Hospital	No previous mental illness	Moderate	13	16.5 (0.9)	15–18	Not reported
Burckhardt et al. (38)	RCT	School	General population	Moderate	338	14.7	Not reported	58.3
Calear et al. (39)	RCT	School	General population	Mild	1477	14.3 (0.8)	12–17	55.9
Carrasco (40).	Single Cohort	Mental health clinic	Depression	Mild-mod	15	Not reported	12–18	100
Chapman et al. (41)	Single cohort	Mental health clinic	Depression, anxiety	Mod-severe	11	14.7	13–16	63.6
Chen et al. (42)	Single cohort	Mental health clinic	Major Depressive Disorder or Autism	Mod-severe	835	Not reported	Not reported	Not reported
Clarke et al. (43)	RCT	Health maintenance organization	Depression	Mod-severe	160	22.7 (2.5)	18–24	80
de Voogd et al. (44)	RCT	School	General population	None	168	14.4 (1.16)	11–18	50.5
Gerrits et al. (45)	Single cohort	General community	Depression	Severe	140	19.7 (3.8)	Not reported	81.5
Gladstone et al. (46)	Single cohort	Primary care	Depression	Moderate	83	17.5 (2.0)	14–21	56.2
Hetrick et al. (47)	RCT	School	Suicidal ideation, self-harm	Severe	50	14.7 (1.4)	13–19	41
Ip et al. (49)	RCT	School	Depression	Mild-mod	257	14.6 (0.8)	13–17	68.1
King et al. (50)	RCT	College	Suicide risk	Severe	76	22.9 (5.0)	>18	59.2
Kramer et al. (51)	RCT	General community	Depression	Mod-severe	263	Not reported	12–22	78.7
Levin et al. (52)	RCT	College	General population	Mild	76	18.4 (0.5)	18–20	53.9
Lillevoll et al. (53)	RCT	School	General population	N/A	1337	16.8 (1.0)	15–20	50.5
Manicavasagar et al. (54)	RCT	Schools & Youth organisations	General population	N/A	235	15.4 (1.7)	12–18	67.5
Melnyk et al. (55)	RCT	College	General population	Moderate	121	18.6	Not reported	86.4
Merry et al. (56)	RCT	Primary care	Depression	Mild-mod	188	15.6	12–19	64.8
Neil et al. (57)	RCT	Schools, community	General population	None-mild	8,207	Not reported	13–19	60
Pinto et al. (58)	RCT	Community	Depression, anxiety	Not reported	60	22 (2.5)	18–25	67
Reid et al. (59)	RCT	Primary care	Emotional/mental health issue	Mild-severe	114	18 (3.2)	14–25	71.5
Rice et al. (60)	Single cohort	Youth mental health clinics	Depression	Severe	42	18.5	15–24	50
Rickhi et al. (61)	RCT	Community	Major Depressive Disorder	Mild-mod	62	18.1	13-24	71
Robinson et al. (62)	Single cohort (pre-post design)	Schools, youth mental health clinics	Suicidal ideation	Severe	32	15.6	14–18	90.5
Robinson et al. (63)	Single cohort (pre-post design)	Schools, youth mental health clinics	Suicidal ideation	Severe	21	15.7	14–18	90.5
Saulsberry et al. (64)	RCT	Primary care	Depression	Persistent, subthreshold	82	17.3 (1.9)	Not reported	57
Sekizaki et al. (65)	RCT	Schools	General population	Mild	80	Not reported	Not reported	0
Smith et al. (66)	RCT	Schools	Depression	Mild-mod	112	Not reported	12–16	Not reported
Spence et al. (67)	RCT	Unclear	Anxiety	Severe	115	14 (1.6)	12–18	59.1
Stasiak et al. (68)	RCT	Schools	Depression	Mild-mod	34	15.2 (1.5)	13–18	41.2
Taylor-Rodgers & Batterham (69)	RCT	University	General population	Mild	67	21.9 (2.0)	18–25	74.7
van der Zanden et al. (70)	RCT	Mental health care	Depression	Mild-severe	144	20.9 (2.3)	16–25	84.5
Wade et al. (71)	RCT	Hospital	People with traumatic brain injury	Moderate	41	Not reported	11–18	Not reported
Whiteside et al. (72)	Case studies	Health clinic	Anxiety & Obsessive-compulsive disorder	Mild	2	13	10–16	50
Whittaker et al. (73)	RCT	Schools	General population	Not reported	855	14	13–17	68.3% female
Whittaker et al. (74)	RCT	Schools	General population	Mild	855	14	13–17	68% female
Wojtowicz et al. (75)	Single cohort	University	Depression, anxiety, stress	Mild-mod	65	23.2 (5)	Not reported	86.2

Studies included participants with no specific mental health symptoms at baseline (n = 12), some with varying levels of depression (mild to moderate n = 7, moderate to severe n = 3, severe = 1, all levels n = 6), others with diagnosed MDD (n = 2) or suicidal risk (n = 5). Participants with varying levels of anxiety were also a focus in some studies (mild n = 1, mild-mod n = 2, mod to severe n = 1, severe n = 1, all levels n = 1). Two studies looked at people with a variety of mental illnesses, and another focused on mood issues in people with traumatic brain injury (**Table 1**).

### Intervention Characteristics

Overall, 32 different DMHIs were investigated across the 41 papers (**Table 2**). Several DMHIs were evaluated in multiple studies including Bite Back (n = 2), CATCH-IT (n = 2), Master Your Mood (n = 2), MoodGym (n = 3), Reframe-IT (n = 3), SPARX (n = 2), and MEMO (n = 2). Several of these papers reported results from the same data sets (62, 63, 73, 74), but reported on different aspects of the study and therefore both papers were included in the review. Most of the DMHIs drew on established therapeutic models, primarily Cognitive Behavioural Therapy (CBT) (n = 28) or a combination of CBT with other models.

**Table 2:** Intervention Characteristics.

Paper	Program Name	Type of technology	Intervention type	Modules	Programme access setting	Personal interaction during programme completion
Anstiss and Davies (35)	Reach Out, Rise Up	Text-messages	CBT	Psychoed messages, weekly challenges, inspiring messages	Own time	Could access trained support
Bobier et al. (36)	SPARX	Computer game	CBT	Challenges, puzzles, psycho-education on mood management	Hospital	Minimal supervision from health professional; reminders giver
Bradley et al. (37)	The Feeling Better program	Online program	CBT	Online learning modules	Hospital	None
Burckhardt et al. (38)	Bite Back	Online program	Positive psychology	Interactive activities, workbook	School	Moderation of posts by therapist
Calear et al. (39)	MoodGYM	Online program	CBT	Online learning modules and exercises	School	Programme presented by classroom teacher
Carrasco (40).	Maya	Video game	CBT & interpersonal psychology	Game in which participants had to make decisions and were given feedback	Own time	None
Chapman et al. (41)	Pesky gNATs	Video game and Mobile App	CBT	Game to coach mindfulness and self-regulation skills, relaxation and mindfulness activities	Clinic	Delivered by a psychologist
Chen et al. (42)	EpxDepression	Phone calls and text messages	Referral to care	Phone-based prompts to record mood; referred to care team if high clinical symptoms	Own time	None
Clarke et al. (43)	[Unnamed]	Online program	CBT	Mood ratings; information pages; journal; interactive tutorials	Own time	Reminders sent
de Voogd et al. (44)	EmoWM	Online program	Emotional working memory	Training tasks to improve working memory in the context of emotional information	School	Initial training at school
Gerrits et al. (45)	Master Your Mood	Online course & chat group	CBT	Course materials and online chat	Own time	Online chat facilitated by health professional; reminders sent to complete materials
Gladstone et al. (46)	CATCH-IT	Online program	CBT, behavioural vaccine model.	Online learning modules; parent workbook	Clinic	Physician interviews
Hetrick et al. (47)	Reframe-IT	Online program	CBT	Online learning modules delivered *via* a series of video diaries and activities	School	Programme presented by school wellbeing staff
Horgan et al. (48)	www.losetheblues.ie	Online forum	Peer support	Peer support forum and online materials	Own time	None
Ip et al. (49)	Grasp the Opportunity (Modified from CATCH-IT)	Online program	CBT	Online learning modules	Own time	Monthly phone call reminders
King et al. (50)	eBridge	Online chat	Motivational Interviewing	Online chat with counsellor	Own time	Online chat with counsellor
Levin et al. (52)	ACT-CL	Online program	Acceptance and commitment therapy	Multimedia lessons; custom emails	Own time	None
Lillevoll et al. (53)	MoodGYM	Online program	CBT	Online learning modules and exercises	Own time	Weekly email reminders sent
Manicavasagar et al. (54)	Bite Back	Online program	Positive psychology	Online interactive exercises	Own time	None
Melnyk et al. (55)	COPE	Online program	CBT	Online learning modules	College	Completed as part of compulsory course
Merry et al. (56)	SPARX	Computer game	CBT	Challenges, puzzles, psycho-education on mood management	Own time	None
Neil et al. (57)	MoodGYM	Online program	CBT	Online learning modules and exercises	One group at school; one group in own time	School group completed it during a designated class period under supervision of classroom teacher
Pinto et al. (58)	eSMART-MH	Computer game	CBT	Avatar based game for practicing communicating about symptoms	Lab	None
Reid et al. (59)	Mobiletype	Mobile App	Referral to care	Self monitoring by assessing 8 areas of functioning	Own time	None
Rice et al. (60)	Rebound	Online program	Moderated Online Social Therapy (MOST)	Online social networking; individually tailored psychosocial interventions; expert and peer moderators	Own time	Ongoing access to clinical moderator; peer discussions
Rickhi et al. (61)	LEAP Project	Online program	Spiritual health	Online learning modules	Own time	None
Robinson et al. (62)	Reframe-IT	Online program	CBT	Online learning modules delivered *via* a series of video diaries and activities	School	Mood ratings checked weekly; message board moderated; completed in presence of research team
Robinson et al. (63)	Reframe-IT	Online program	CBT	Online learning modules delivered *via* a series of video diaries and activities	School	Mood ratings checked weekly; message board moderated; completed in presence of research team
Saulsberry et al. (64)	CATCH-IT	Online program	CBT	Online learning modules; parent workbook	Own time	Interviews with physician or research tteam
Sekizaki et al. (65)	[Unnamed]	Online program	CBT	Online group education and online homework	School	Completed in class groups
Smith et al. (66)	Stressbusters	Computer program	CBT	Interactive multimedia, activities, diaries, worksheets	School	Completed individually during school hours with up to 4 other students in a room
Stasiak et al. (68)	The Journey	Computer program	CBT	Learning modules presented in game-like environment; interactive exercises	School	Some supervision by school counselor
Taylor-Rodgers & Batterham (69)	[Unnamed]	Online program	Psychoed	Psychoeducation; vignettes	Own time	None
van der Zanden et al. (70)	Master Your Mood	Online group course	CBT	Delivered in online chat room using text and images; homework	Own time	Delivered by professional mental health promotion workers
Wade et al. (71)	TOPS	Online program	Problem-solving	Online learning modules, videoconferences	Own time	Delivered by psychologist and psychology students
Whiteside et al. (72)	Mayo Clinic Anxiety Coach	Mobile App	CBT	Assessment, psychoeducation & treatment	Own time	Minimal contact with therapist
Whittaker et al. (73)	MEMO	Mobile MMS	CBT	Mobile phone messages containing text, video, cartoon messages and a mobile website	Own time	None
Whittaker et al. (74)	MEMO	Mobile MMS	CBT	Mobile phone messages containing text, video, cartoon messages and a mobile website	Own time	None
Wojtowicz et al. (75)	[Unnamed]	Online program	Theory of planned behaviour, CBT	Online learning modules	Own time	Contacted by program coach weekly

The technologies utilized in the various DMHIs included some phone-based interventions such as text-messages (n = 4) and smartphone applications containing assessment tools and/or psychoeducational materials (n = 3). The majority of DMHIs were web-based (n = 30), including many with online modules, learning materials or activities (n = 24), group chats or courses (n = 2), online forums (n = 2), and online chat facilities with a mental health professional (n = 2). Others were computer-based but not online, including games (n = 5) and psychoeducational computer programs (n = 2).

Many DMHIs included learning modules (n = 18), interactive learning activities (n = 6), psychoeducational materials in a variety of formats including text and video (n = 7), or game-based learning activities (n = 4). Additional features included regular inspiring messages (n = 1), challenges (n = 3), mood tracking (n = 3), or diary/journals (n = 2). Four studies included DMHIs with an accompanying workbook for participants or their parents. Only 11 of the studies reviewed included DMHIs that were entirely self-help and were completed in the participant’s own time. The rest of the studies involved interaction with a mental health professional or completion of the intervention in some kind of supervised setting. Two of these were completed in hospitals, one with some minimal supervision from a health professional (36). Others were completed in a school setting (n = 10). Some of the studies completed at school involved a high level of supervision (n = 5) such as in studies where the intervention was presented by the school wellbeing staff (47), the classroom teacher (39), in the presence of the research team (62, 63), or in class groups (65). Other school-based studies involved lower levels of interaction with a therapist or the research team (n = 3) such as moderation of an online discussion board by a therapist (38), initial training completed at school but the intervention otherwise used in the students’ own time (44), and where the intervention was accessed at school with minimal supervision by the school counselor (68). Studies outside of school settings included DMHIs that could be completed at home in the participant’s own time, but included interactions with a therapist such as sending reminders or text messages (n = 4), or participating in online group courses or chats (n = 9).

### Effectiveness of DMHIs

**Figure 2:**
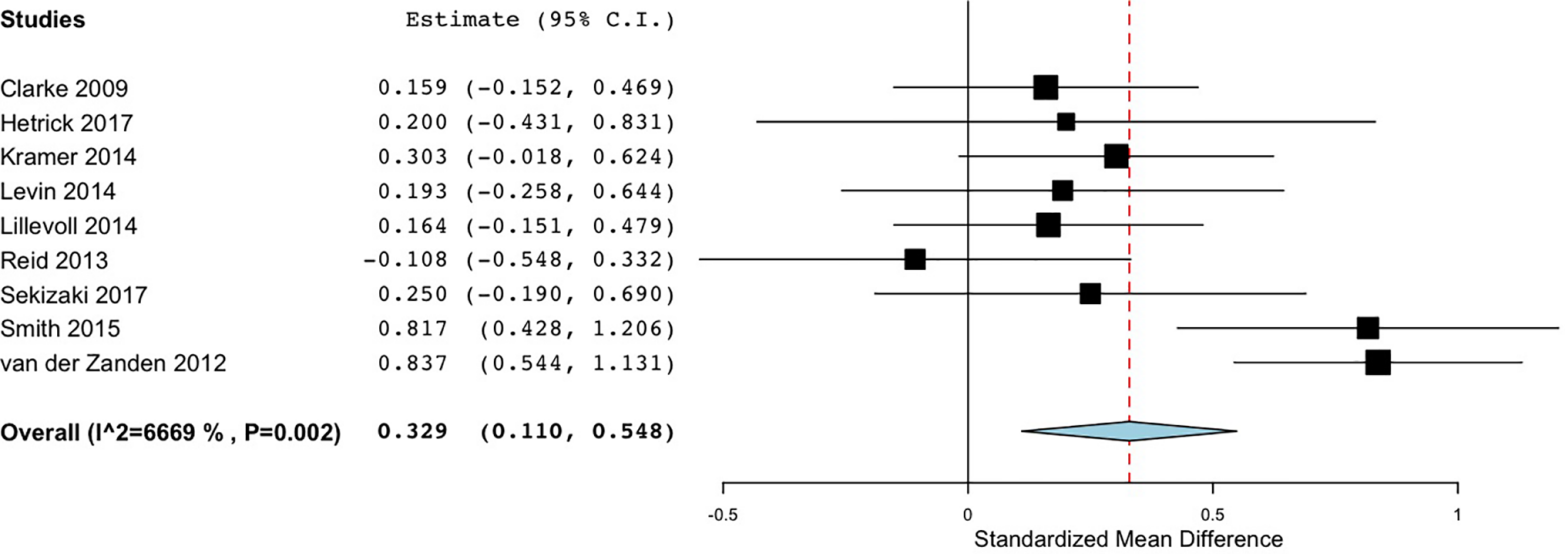
Forest plot of meta-analysis of randomised controlled comparisons between DMHIs and no intervention for depression in adolescents.

**Figure 3:**
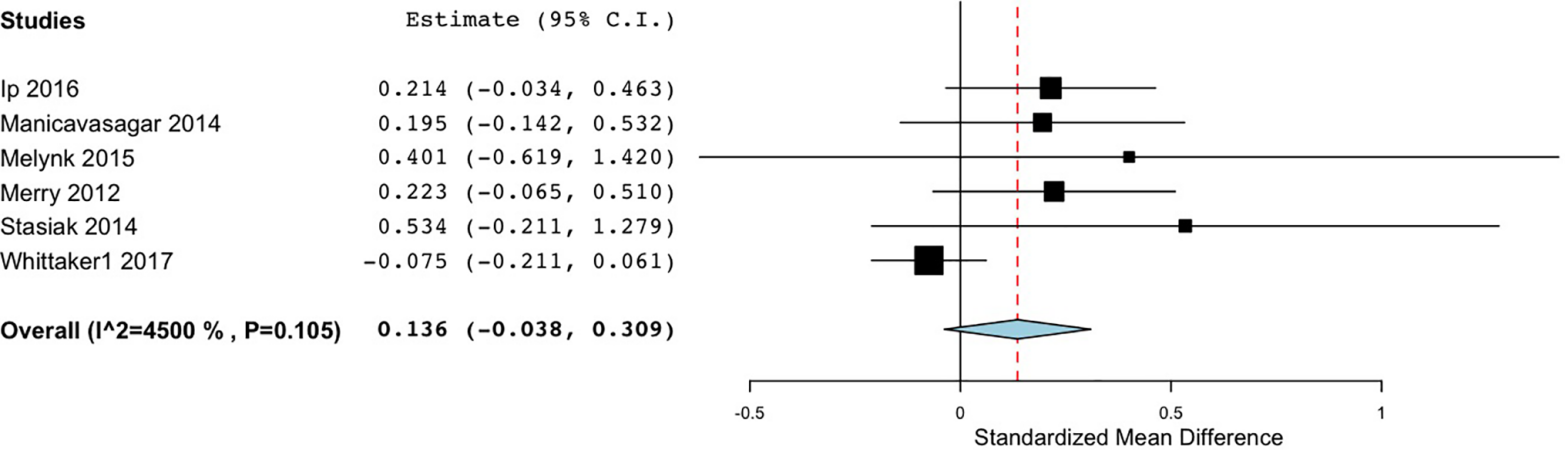
Forest plot of meta-analysis of randomised controlled comparisons between DMHIs and active control groups for depression in adolescents.

The effectiveness of various DMHIs in treating symptoms of depression was compared to a control group in 15 studies (**Table 3**). Nine of these studies compared DMHIs to no intervention (a waitlist control group), while five of the studies compared DMHIs to an active control in which some alternative online materials were used, including one with some psycho-educational content (68), and one contained a Treatment As Usual (TAU) comparison group in which face-to-face counseling was offered (56). Since the TAU group in this case included active treatment it was combined in analysis with the active control groups. The pooled effect size of studies comparing the intervention group to a no-intervention group (n = 9) was 0.33 (95% CI 0.11 to 0.55) (**Figure 2**), suggesting that DMHIs have a small effect size when compared to a no intervention control group, while the pooled effect size of studies comparing the intervention group to an active control group (n = 6) was 0.14 (95% CI -0.04 to 0.31). Thus this review did not find a difference in outcomes between DMHIs and active controls, including a mixture of usual care for depression and non-depression specific interventions (**Figure 3**). Heterogeneity was relatively high (*I^2 =^* 70%) and statistically significant (p < .001). Two studies had negative effect sizes indicating that the control group had lower depression scores at post-test than the intervention group (59, 74). Reid and colleagues (59) evaluated the effectiveness in comparison to a waitlist control group of a smartphone application that allowed self-assessment on 8 domains of mood and functioning, referring this information to general practitioners for medical review. No significant effect on depression was found at post-test, but increased emotional self-awareness was reported. Whittaker and colleagues (74) similarly used a phone-based approach, delivering multimedia messages based on CBT and comparing this to use of similar multimedia messages with no focus on depression. The authors concluded that there was no evidence of benefits superior to the active comparison program with content about healthy behaviours.

**Table 3:** RCTs included in meta-analysis.

Paper	Level of interaction	Sample size	Control Group	Outcome Measure	Effectiveness Effect Size (Cohen’s *d*)	Confidence Interval
Clarke et al. (43)	L	I = 83, C = 77	Wait list	PHQ	0.16	-0.15 to 0.47
Hetrick et al. (47)	H	I = 26. C = 24	Wait list	RADS	0.20	-0.43 to 0.81
Ip et al. (49)	L	I = 130, C = 127	Antismoking website	CES-D	0.21	-0.03 to 0.46
Kramer et al. (51)	H	I = 131, C = 132	Wait list	CES-D	0.30	-0.02 to 0.62
Levin et al. (52)	N	I = 37, C = 39	Wait list	DASS	0.19	-0.26 to 0.64
Lillevoll et al. (53)	L	I = 42, C = 483	Wait list*	CES-D	0.25	-0.23 to 0.72
Manicavasagar et al. (54)	N	I = 120, C = 115	Alternative websites	DASS	0.20	-0.14 to 0.53
Melnyk et al. (55)	H	I = 82, C = 39	Introductory content about university	PHQ	0.40	-0.62 to 1,42
Merry et al. (56)	N	I = 94, C = 94	TAU	CDRS-R	0.22	-0.07 to 0.51
Reid et al. (59)	N	I = 68, C = 46	Wait list	DASS	-0.11	-0.55 to 0.33
Sekizaki et al. (65)	H	I = 40, C = 40	Wait list	K6	0.25	-0.19 to 0.70
Smith et al., (66)	H	I = 55, C = 57	Wait list	MFQ	0.82	0.43 to 1.21
Stasiak et al. (68)	H	I = 17, C = 17	Alternative online program including psycho-educational content	CDRS-R	0.53	-0.21 to 1.28
van der Zanden et al. (70)	H	I = 121, C = 123	Wait list	CES-D	0.84	0.54 to 1.13
Whittaker et al. (74)	N	I = 418, C = 417	Alternative material	CDRS-R,	-0.08	-0.21 to 0.06

PHQ, Patient Health Questionnaire; DASS, Depression, Anxiety, Stress Scale, RADS, Reynolds Adolescent Depression Scale; CDRS-R, Children’s Depression Rating Scale Revised; K6, Kessler 6; CES-D, Centre for Epidemiological Studies Depression Scale.

* The study included active comparison groups as well, but only the comparison to the waitlist control group was included in this analysis.

C, control group; H, High level of interaction; involved direct contact with a therapist or were completed in supervised settings; I, Intervention group; L, Low level of interaction; limited interaction such as regular emails, text messages or optional opportunities to contact a therapist; N, No interaction; did not involve any interaction with a mental health professional and were completed unsupervised in personal time.

Sub-group analyses were conducted to investigate the effect of therapist interactions and study completion settings on the outcomes in the 9 studies that compared the intervention to no intervention (**Table 3**), and the 6 studies comparing the intervention to an active control group. Studies were categorised as having High levels of human interaction (H) if they involved direct contact with a therapist or were completed in supervised settings such as a lab, clinic or school (47, 51, 55, 65, 66, 68, 70). They were categorised as Low interaction (L) (43, 49, 53) if they had some limited interaction such as regular emails, text messages, or optional opportunities to contact a therapist, or No interaction (N) (52, 54, 56, 59, 74) if they did not involve any interaction with a mental health professional and were completed unsupervised in personal time.

**Figure 4:**
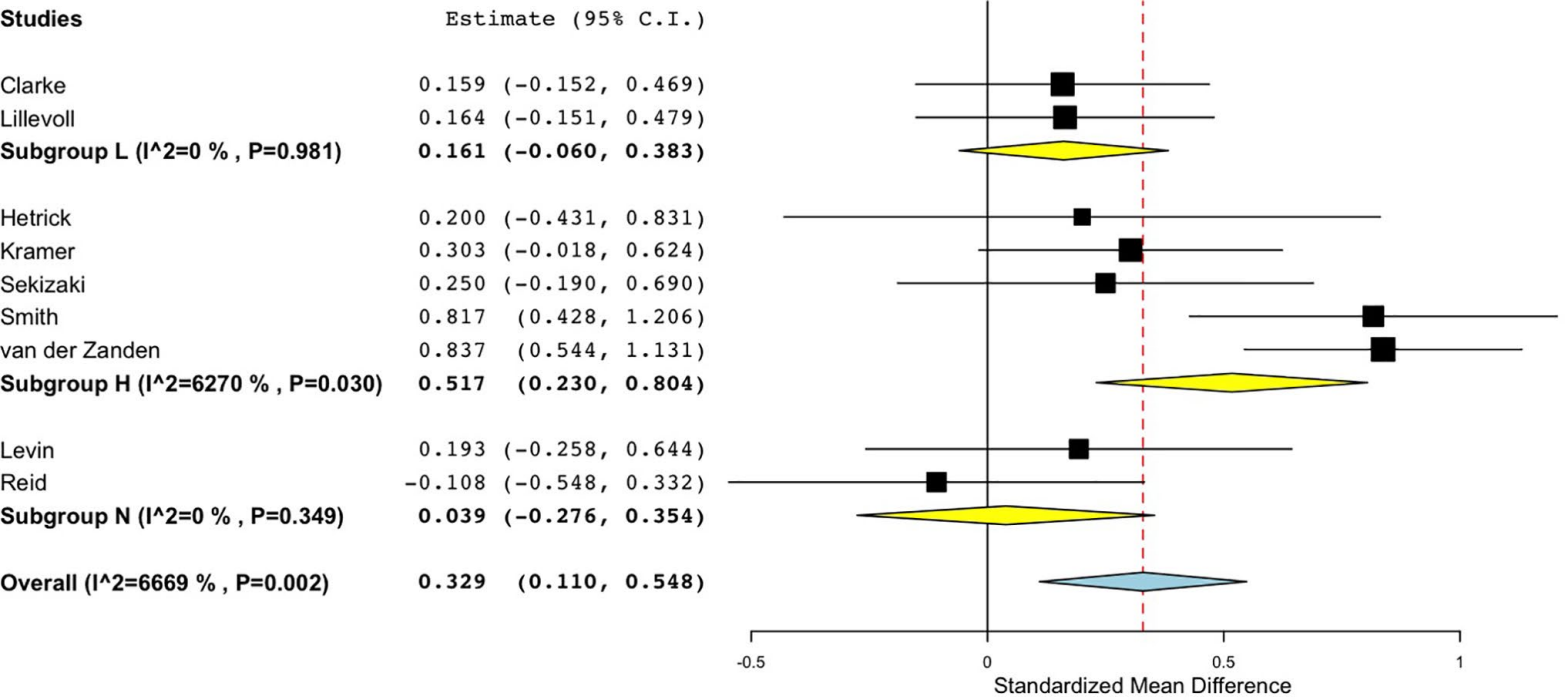
Forest plot of sub-group analysis for randomised controlled comparisons between DMHIs with high, low or no support compared to no intervention for depression in adolescents.

For studies comparing the DMHI to no intervention, the pooled effect size was smallest in the No interaction group (*d* = 0.04), and also small in the Low interaction group (*d* = 0.16), while the High interaction group returned a medium effect size (*d* = 0.52) (**Figure 4**). This indicates that DMHIs were mostly effective when they involved high levels of human interaction. The DMHIs in the No interaction group that did have a positive effect size were highly interactive, containing multimedia lessons (52), interactive online exercises (54), and game-based challenges and puzzles aiming to improve mental health literacy (56). Levin and colleagues (52) did not include direct conversations with mental health professionals. However, the system did send automatically generated emails that were customized based on participants’ earlier input. This could have given the illusion of human interaction, thereby increasing the effectiveness of the program despite there being no direct personal communication. No significant differences were found according to intervention type or severity of symptoms at baseline.

**Figure 5:**
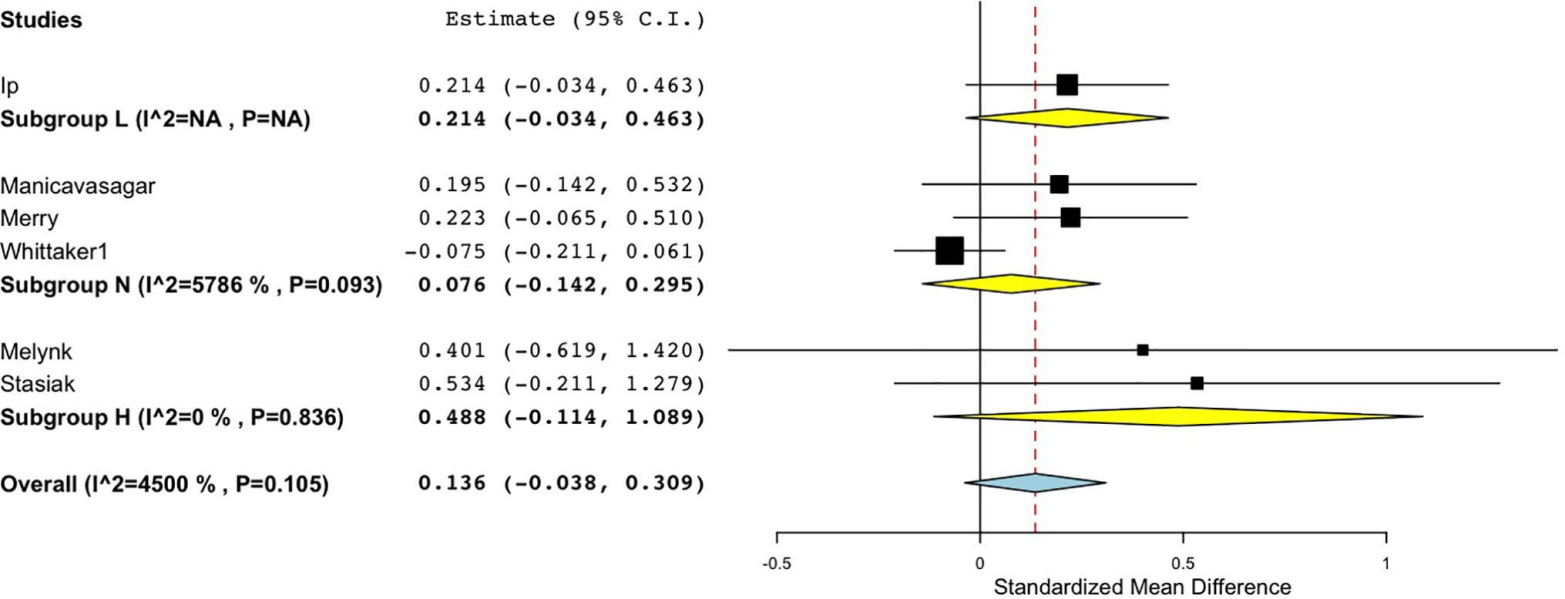
Forest plot of sub-group analysis of Interaction level for randomised controlled comparisons between DMHIs with high, low and no support compared to active control groups for depression in adolescents.

Similarly, for the studies with active comparison groups, the pooled effect size was again smallest in the No interaction group (*d* = 0.08), and also small in the Low interaction group (*d* = 0.21), while the High interaction group returned a medium effect size (*d* = 0.49) (**Figure 5**). Both of the studies in the High interaction group were completed in school classroom settings (55, 68). Thus, across both active and no intervention control groups, effect sizes reached a moderate size only when there was a high level of therapist interaction or supervision in the study design.

### Risk of Bias

The proportion of studies at high/unclear risk of bias was: 34% selection bias (e.g. randomization or allocation concealment), 63% detection bias (e.g. blinding of outcome assessment), 41% attrition bias, and 31% selective reporting (**Table 4**). Overall 76% of the studies included in the review as a whole, and all but 4 studies (43, 66, 68, 74) included in the meta-analysis had some risk of bias. Sub-group analyses were performed on the 15 studies included in the meta-analyses according to type of bias. Significant differences were found for selection bias with the low risk of bias group having a lower pooled effect size (*d* = 0.01, 95% CI -0.05 to 0.24) than the unclear or high-risk group (*d* = 0.44, 95% CI 0.22 to 0.69). Studies with low risk of detection bias also had a lower pooled effect size (*d* = .09, 95% CI -0.08 to 0.26) than the unclear or high-risk group (*d* = .40, 95% CI 0.18 to 0.62).

**Table 4:** Assessment of bias across all studies

Article4	Selection bias	Performance bias	Detection bias	Attrition bias	Reporting bias	Overall bias
Anstiss and Davies (35)	High	Unclear	Unclear	High	High	High
Bobier et al. (36)	Low	Unclear	Unclear	Low	Low	Unclear
Bradley et al. (37)	Low	Low	Low	Unclear	High	Unclear
Burckhardt et al. (38)	Low	Low	Low	Low	Low	Low
Calear et al. (39)	Low	Unclear	Unclear	Low	Low	Unclear
Carrasco (40).	Low	Low	Low	Low	Low	Low
Chapman et al. (41)	Low	Low	Low	Low	Low	Low
Chen et al. (42)	Low	Low	Low	Low	Low	Low
Clarke et al. (43)	Low	Low	Unclear	Low	Low	Low
de Voogd et al (44)	High	Low	Low	High	High	High
Gerrits et al. (45)	Low	Low	Low	High	Low	High
Gladstone et al. (46)	Low	Low	High	Low	Low	High
Hetrick et al. (47)	Low	Low	High	High	Low	High
Horgan et al. (48)	Low	Low	High	High	High	High
Ip et al. (49)	Low	Low	Low	High	Low	High
King et al. (50)	Low	Low	Unclear	Low	High	High
Kramer et al. (51)	High	Low	High	High	Low	High
Levin et al. (52)	High	Low	High	Low	Low	High
Lillevoll et al. (53)	Unclear	Low	Unclear	High	Low	High
Manicavasagar et al. (54)	Unclear	Low	Unclear	High	Low	High
Melnyk et al. (55)	High	Low	Unclear	Unclear	High	High
Merry et al. (56)	Low	Low	High	Low	Low	High
Neil et al. (57)	Unclear	Low	Unclear	Unclear	High	High
Pinto et al. (58)	Low	Low	High	High	Low	High
Reid et al. (59)	Low	Low	High	Low	Low	High
Rice et al. (60)	Low	Low	Low	Low	Low	Low
Rickhi et al. (61)	Low	Low	High	Low	High	High
Robinson et al. (62)	High	Low	Low	High	High	High
Robinson et al. (63)	High	Low	Low	High	High	High
Saulsberry et al. (64)	Low	Low	High	High	Low	High
Sekizaki et al. (65)	High	Low	High	Low	Low	High
Smith et al. (66)	Unclear	Low	Unclear	Low	Low	Unclear
Spence et al. (67)	Low	Low	High	Low	Low	High
Stasiak et al. (68)	Low	Low	Low	Low	Low	Low
Taylor-Rodgers & Batterham (69)	Low	Low	High	Low	Low	High
van der Zanden et al. (70)	Unclear	Low	Unclear	Low	Low	Unclear
Wade et al. (71)	Low	Low	High	Low	Low	High
Whiteside et al. (72)	High	Low	High	Low	Low	High
Whittaker et al. (73)	Low	Low	Low	Low	Low	Low
Whittaker et al. (74)	Low	Low	Low	Low	Low	Low
Wojtowicz et al. (75)	Low	Low	High	Unclear	High	High

### Attrition, Adherence, and Engagement

Attrition rates were defined as the number of participants who completed the study as a percentage of the participants who commenced the intervention (**Table 5**).Information about adherence and engagement (how much those who completed the study engaged with the intervention), tended to be reported differently across papers. For example, some papers reported module completion rates (36). Others reported time spent on a website (43). Overall 16 (39%) of the studies had attrition rates over 20%, the level broadly considered indicative of possible attrition bias (76) (**Table 5**). In several of these studies, while attrition rates were high, they were equal between groups (for e.g. 50) suggesting that drop out rates related more to recruitment methods than to non-engagement. However, this was not always the case and in many studies, even among those with low study attrition, engagement tended to be low, with participants completing less than half of the intervention components (36, 45, 49, 51, 53, 73). The majority of these studies were again ones that involved completion in one’s own time. For example, Ip and colleagues (2016) reported low drop out rates and a small effect size. However, participants only completed roughly three of 10 modules and spent about 39 minutes on the website over 4 months, suggesting relatively low engagement.

**Table 5:** Attrition rates, sample sizes and indicators of adherence and engagement.

Study	Sample Size at Commencement	Attrition (%)	Indicators of Adherence & Engagement as Reported in Papers
Anstiss et al. (35)	40	45	Two participants opted out after commencing. 16 did not complete post-intervention evaluations
Bobier et al. (36)	20	30	60% did >1 module but did not complete prior to discharge; 10% completed all 7 modules
Bradley et al. (37)	13	NR	NR
Burckhardt et al. (38)	I = 177, C = 161	I = 19, C = 10.6	Two schools withdrew, one due to negative feedback from students. 8% of students didn’t return any workbooks, 55.6% returned 5-6 workbooks.
Calear et al. (39)	1477	NR	15% of participants completed at least 20 of 29 exercises
Carrasco (40).	15	13.3	Average playtime was 11:57 minutes. Most played the game once only. Four people played it twice.
Chapman et al. (41)	11	0	N/A – Completed with clinician
Chen et al. (42)	3	0	100% responded to weekly prompts. Daily responses were lower and decreased over time
Clarke et al. (43)	I = 83, C = 77	I = 20.5, C = 28.2	Median session = 6, Mean (SD) session = 8.5 (14.2), Cumulative mean (SD) time on site = 115.1 mins (176.1)
de Voogd et al (44)	I = 129, C = 39	I = 10.9, C = 5.1	NR
Gerrits et al. (45)	140	64.3	53.6% participated in less than 4 chat sessions, 35.7% finished all 8 sessions.
Gladstone et al. (46)	I (group 1) = 43, I (group 2) = 40	I (group 1) = 16.3, I (group 2) = 17.5	NR
Hetrick et al. (47)	1 = 26, C = 24	I = 30.7, C = 12.5	Average number of modules commenced was 5 out of 8. Seven commenced only 1-2 modules, 8 commenced all modules. Message board used by only 6 participants, 5 of them to discuss technical issues.
Horgan et al. (48)	118	71.2	53 forum posts made by 17 different users over 3 months
Ip et al. (49)	I = 130, C = 127	I = 5.4, C = 0	Median time on website was 39.3 mins, median of 3 of 10 modules completed
King et al. (50)	I = 41, C = 35	I = 24.4, C = 17.1	71% in the intervention group did not correspond with counsellor.
Kramer et al. (51)	I = 131, C = 132	I = 43, C = 42	Mean number of chats = 1.36 (SD 2.08). 58% did not have any chats.
Levin et al. (52)	I = 37, C = 39	I = 5.4, C = 2.6	92% completed both lessons, average of 81.98 mins (SD = 22.68) within 3 weeks. 85.3% reported reading the emails, and 69% of those who read the emails completed the suggested exercises
Lillevoll et al. (53)	I = 42, C = 483	74.3 overall	Only 8.5% of participants signed on and used the intervention
Manicavasagar et al. (54)	I = 120, C = 115	I = 37.5, C = 20	36 participants used the website for < hour a week due to time constraints, technical issues, and website content.
Melnyk et al. (55)	I = 82, C = 39	NR	One participant failed to complete any sessions; the other completed all seven.
Merry et al. (56)	I = 92, C = 93	I = 7.6, C = 8.6	Two participants withdrew due to needing face-to-face assistance for severe symptoms. 86% completed at least 4 modules, 60% completed all modules.
Neil et al. (57)	I (group 1) = 1000, I (group 2) = 7207	NR	Completion rates higher in school-based sample than those in the community-based sample. In the community sample 89% completed none or only one module.
Pinto et al. (58)	I = 30, C = 30	I = 60, C = 46.7	NR
Reid et al. (59)	I = 68, C = 46	I = 23.5, C = 28.6	Average of 3.3 entries per day, completed on average in 14.6 days
Rice et al. (60)	42	7.1	System usage was high with an average of 72.2 logins and 51.1 posts per user.
Rickhi et al. (61)	I = 34, C = 29	I = 23.5, C = 13.8	87% completed the full 8-week project
Robinson et al. (62)	27	22.2	21 participants completed all modules. Reasons given for dropping out included feeling better, changing schools, having too much homework and being too unwell.
Robinson et al. (63)	27	22.2	As above
Saulsberry et al. (64)	I = 40, C = 42	I = 27.5, C = 19.0	NR
Sekizaki et al. (65)	I = 40, C = 40	NR	Only 7 participants accessed the intervention less than 10 times. Average access times over 4 weeks was 16.9
Smith et al. (66)	I = 55, C = 57	I = 0, C = 3.5	86% completed all 8 sessions, 93% completed at least half
Spence et al. (67)	I (group 1) = 44, I (group 2) = 44, C = 27	1 (group 1) = 6.8, I (group 2) = 9, C = 14.8	Average number of sessions completed in E1 was 7.5 out of 10 and 4.48 out of 5 for parents. Only 39% adolescents and 66% of parents completed all treatment sessions.
Stasiak et al. (68)	I = 17, C = 17	I = 5.9, C = 23.5	NR
Taylor-Rodgers & Batterham (69)	I = 33, C = 34	I = 15.2, C = 17.6	65.4% reported viewing all three web-pages
van der Zanden et al. (70)	I = 121, C = 123	I = 21, C = 20	52% attended at least 4 of 6 sessions. Only 20% attended all.
Wade et al. (71)	I = 20, C = 20	I = 20, C = 5	NR
Whiteside et al. (72)	2	0	NR
Whittaker et al. (73)	I = 426, C = 429	I = 1.9, C = 2.8	74.4% viewed at least half the messages, 29.6% viewed all or most.
Whittaker et al. (74)	I = 426, C = 429	I = 1.9, C = 2.8	Majority said they had read at least half the messages, but data from the messaging gateway showed that only 19% actually saw at least half the messages.
Wojtowicz et al. (75)	I (group 1) = 24, (I group 2) = 24, C = 17	NR	NR

C, Comparator group; I, Intervention group; NR, Not reported.

Qualitative data from all papers were categorized according to features of the DMHIs that were liked by participants, features that were disliked (**Table 6**), and features predicting adherence. There were four key categories of data in relation to liked features. The first category related to ***social support***. Several studies reported that participants had found it useful to be in contact with professionals. Participants in one study who had access to a trained supporter in addition to regular text messages reported that they “liked talking to someone who was friendly” (35, p. 101). Participants in one study of a game-based CBT intervention called Pesky gNATs (41) reported liking the fact that doing it on a computer was “not as full on as face-to-face” (p. 15). However this was not perceived by all to be preferable to in-person contact. For example, two studies that evaluated an interactive CBT-based fantasy game called SPARX, both found that some participants preferred face-to-face support from a therapist (36, 56). Similarly, some participants in an online CBT-based group course reported a preference for a face-to-face version of the course (45).

**Table 6:** Liked and disliked features of DMHIs.

Liked Features	Disliked Features
Social Support: • With professionals • With peers	Preference for real contact
Online or computer-based: • Privacy and anonymity • Fits into daily routine; feels normal • Go at own pace • Accessibility • Fun, relaxing, distracting	Content that is too juvenile or patronising
Useful content: • Problem solving and anger control • Time management and challenging negative thoughts • Relaxation and coping with stress • Acceptance • About mental health generally	Educational materials: • Boring/less engaging • Hard work • Repetitive • Need for personalisation
Look and feel: • Relatable • Interactive/game-like • Video components • Aesthetically appealing • Easy to use and navigate	Look and feel: • Colour scheme • Lack of variety • Customisation needed • Technical glitches or difficulties navigating sites

For other participants, it was the opportunity to connect with peers who were experiencing similar difficulties that was helpful. Gerrits and colleagues (56) reported that participants “found chatting to be a pleasant and positive way to talk about being down and their feelings of depression” (p. 6). Similarly, Horgan and colleagues (48) studied the impact of an online forum and reported that participants found it “good to say what was going on aloud (albeit in writing)” (p. 87). One participant in the same study stated: “Its about empathy and the realization that you’re not alone. That others are feeling the same way you do and are having trouble coping” (p. 87).

For some participants the primary attraction of DMHIs were their ***online or computer-based*** nature. In contrast to users noted above who reported a preference for face-to-face contact, for many users a key benefit of DMHIs was the privacy they afforded. One participant in an online self-help program, Crystal, stated: “You can kind of do it in a secluded area where nobody is watching you … the privacy is kind of like a really big appeal” (37, p. 28). In one study of an online peer-support website, anonymity allowed participants to share details that they had never shared before, and in fact had “put a lot of effort into hiding” (48, p. 87). DMHIs also had the advantage of fitting into the daily routines of users, connecting with current interests (40), and helping “to bring back a sense of normality” (36, p. 290). A participant named Rob stated: “Most teens are always on the internet … while you’re on say Facebook or something, you can just open up another tab” (37, p. 28) It can also be accessed from a variety of locations such as school, home or in a clinic and participants could “learn by myself and at my own pace” (56, p. 7). Participants reported that it was “fun to be able to do it on a computer” (41, p. 13).

Other participants commented on particularly ***useful content***. Participants reported that the DMHIs “showed me things I didn’t know” (56, p. 7), and helped them learn more about mental health (58). Appreciation was expressed for content that helped participants to learn specific techniques such as problem solving and anger control (71), or challenging negative thoughts (37).

Another major category of the data related to the ***look and feel*** of the DMHIs. Participants preferred situations, characters, or avatars that were relatable. For example, participants reported that it was helpful when the focus was “situations any teenager goes through” such as school and interpersonal relationships (37, p. 27). Conversely, several studies reported that drop-outs occurred when the content “did not seem relevant for them” (54, p. 7). Other features that participants reported liking included interactive activities (58, 62), and video components (62, 73). Similar comments were made in relation to DMHIs with a game-like feel. Participants stated that this made engaging with the DMHIs fun (36, 41). It was also important to the users that DMHIs were aesthetically appealing and easy to use and navigate.

One of the most prominent features that participants reported disliking was the ***educational content*** of many DMHIs. For example in their case study of use of a smartphone app for anxiety, Whiteside and colleagues (72) reported that the participant “appeared less engaged and interested in the background educational content” (p. 86). Multiple other studies reported similar comments by participants, particularly non-completers (36, 45, 54, 56, 58, 73). Educational modules were viewed as too long (37), “tough and sometimes quite tiring” (45, p. 6), “tedious and laborious” (38, p. 6). Some participants argued that it would be more convenient to be able to tailor modules to one’s own needs: “I didn’t like that you couldn’t skip out of something if you already understood the concept”(58, p. 163). Burckhardt and colleagues (2015) suggested that more structured settings and dose effects may have contributed to their negative results, since other studies have found that the number of activities participants are required to do can reach a saturation point (77).

A noteworthy point was that numerous participants reported that the DMHIs often felt ***too juvenile or patronising***. Participants did not enjoy using DMHIs that seemed like they were designed for younger children (41, 68). One participant suggested: “make it more grown up” (41, p. 14).

***Technical glitches and difficulties navigating sites*** were also frequently cited as reasons for low adherence and engagement (37, 54, 68, 73). Participants stated that DMHIs should be improved to make them “comparable to commercially available games” (36, p. 290). Others reported disliking particular ***aesthetic features*** such as the colour scheme and a lack of variety of icons, cartoons, and diagrams (37).

***Factors predicting adherence*. **Only four studies reported ***predictors*** of adherence to the DMHIs. Neil and colleagues (57) compared a school-based completion setting for MoodGYm to a community setting, finding that a school-based setting predicted greater adherence. Gender was also a consistent predictor of adherence, with females being more likely to complete compared to males (51, 57, 70). Mental health also played a role, with higher pre-test scores in depression (57), a longer history of mood disorders (51), or low scores in anxiety at pre-test (70) predicting greater adherence.

## Discussions

This review aimed to determine the types of DMHIs that are effective in treating depression and anxiety in young people and the components of these interventions most associated with positive outcomes and engagement. Overall, studies in relation to depression demonstrated a small effect size in favour of DMHIs when interventions were compared to no intervention. While this might not always reflect a clinically significant level of change, it suggests that such DMHIs may be of value in the context of public health and preventative interventions. On the other hand, studies comparing DMHIs to active control conditions were not effective. In fact, in two studies the control group actually had lower depression levels at post-test than the intervention group. Both studies included phone based interventions: an app that referred users for medical review, and a program of multimedia mobile phone messages. However, given that there was a risk of bias in many studies included in the meta-analyses, these results should be interpreted with caution. In fact, the two studies in the meta-analysis that reported negative effect sizes were two of the only three studies assessed as having low risk of bias. Studies which did not involve blinding of either group allocation or of outcome assessment tended to have higher effect sizes than studies with low risk of bias, indicating that methodological limitations of the studies reviewed likely inflated the larger effect sizes.

Of further importance in our findings was the fact that only DMHIs involving regular interactions with a therapist or that were completed in a supervised setting reached a moderate effect size in comparison to a no-intervention control group, while DMHIs that involved educational programs completed in the participant’s own time were not found to be effective in this study. This suggests that currently available DMHIs may not be effective in causing clinically detectable levels of change unless they involve a high level of supervised use or therapist involvement. These results reflect the overarching significance of human interaction in psychological interventions (78). However, the preference among some participants for human contact revealed in this review existed in tension with the need for privacy and anonymity, suggesting that there is a need for more effective design of DMHIs to fill a gap that traditional face-to-face therapies do not.

Despite this, adherence and engagement rates tended to be low in many studies particularly those where interventions were completed in their own time. This reinforces the idea that many DMHIs are most likely to be useful for people already receiving mental health support or at least those not averse to doing so. However, DMHIs completed in settings such as schools, labs, or clinics cannot reliably indicate their effectiveness in reaching young people outside of these settings. Where DMHIs were completed in their own time or did not include interaction with a mental health professional, effectiveness was much reduced. Nevertheless, for many users it is the anonymity and privacy afforded by the online context that holds the greatest appeal. Therefore, there is a need to balance the competing advantages of anonymity and social support. Other studies have similarly concluded that social networking features in DMHIs are a “gamble” due to the potential for both negative and positive effects (79).

These results indicate two distinct needs in DMHI development. Firstly, a dire need exists to increase the appeal of DMHIs so as to reach the 80% of young people who are not already obtaining professional help. These young people may not understand that their symptoms indicate the need for mental health assistance. Other barriers such as a lack of energy or motivation to engage with complex tasks, or a fear of the stigma of mental illness may prevent them from accessing even DMHIs where such are overtly about mental health or are educational in nature. Research indicates that young men are particularly unlikely to receive professional help (80), which makes the findings in this review that males are less likely to engage with DMHIs than females particularly disturbing. The development of DMHIs that build on the existing interests of young people in non-confronting ways may be of more appeal to this group. DMHIs that particularly cater to the interests of young men are especially needed. Only highly interactive DMHIs involving multimedia materials or game-based activities were successful in studies with low levels of human interaction, suggesting that these types of features are highly appealing.

Secondly, for DMHIs designed to help young people who are already seeking or willing to seek professional assistance but prefer to do so in the relative anonymity of digital settings, it is clear that the help of schools and mental health professionals is a crucial part of the roll out of such interventions. Similarly, some scholars have recommended a model of ‘supportive accountability’, in which accountability to a supporter or coach can enhance adherence to eHealth interventions (81).

The need for further refinement to available DMHIs was confirmed by our qualitative analysis which revealed factors involved in high drop out rates and low engagement rates in numerous studies. In general, participants liked the online or computer based formats and game-like feel of some DMHIs, particularly when the content was interactive, had relatable situations or characters, and had appealing aesthetic features. However, feedback by study participants or people who withdrew from the studies referred to the boring nature and hard work involved in the online learning modules. Participants found DMHIs with non-appealing interfaces, frequent technical glitches, or material that seemed too juvenile to be off-putting. Other studies have similarly found that making DMHIs easy to use and to navigate is important to users (82), and in fact criteria like this are commonly used to evaluate usability and appeal (83).

Although some participants seemed to appreciate the opportunity to learn various psychological skills to improve their wellbeing, many disliked the high educational focus of the interventions, the fact that they seemed designed for children much younger, and that they did not match commercially available programs in quality. This highlights the need for future DMHIs to consider the opinions of young people closely in their design. There is a need for feedback from young people and co-design methods to ensure that both content and aesthetics are appealing to the target audience. There is also a need for DMHI developers to ensure that materials are not ‘dumbed down’ and that they are presented in a way that does not feel like hard work but that builds on the natural interests of young people. This again presents a challenge for developers, to balance the need for simplicity of use with age-appropriate content. This is especially important for DMHIs designed to address depression and anxiety since these conditions are associated with both a lack of motivation (84) and impaired concentration (85), making such users a particular challenge to engage.

The current study was limited by the search terms used. Future reviews should also include search terms such as “internet-delivered”, “computer”, or “computerised”, since this could have picked up a broader range of studies in the current review. Nevertheless, this review demonstrates that while somewhat effective for those who use them, DMHIs fail to appeal to a large proportion of young people. In fact, when compared to active comparison groups including online materials with no psycho-educational content, DMHIs had only minimally better effects. As yet there seems to be a dearth of DMHIs that are likely to attract the large numbers of young people with mental illness who are not already open to receiving professional help. There is thus a need for urgent attention to developing high quality DMHIs that address the weaknesses and focus on the strengths identified, to help young people currently in the shadows to access appealing and accessible tools for managing their mental health. There is also a need for methodologically robust double-blinded RCTs to be designed to provide more stringent testing of the effectiveness of such interventions.

## Data Availability Statement

The datasets generated for this study are available on request to the corresponding author.

## Author Contributions

All authors contributed to the conception and design of the review. SG, CM, and DC were involved in searches, quality assessment and data extraction. SG and CM conducted the analyses. All authors contributed to manuscript revision and read and approved the submitted version.

## Conflict of Interest

The authors declare that the research was conducted in the absence of any commercial or financial relationships that could be construed as a potential conflict of interest.
