# Supplementary material for: What Works and What Doesn’t Work? A Systematic Review of Digital Mental Health Interventions for Depression and Anxiety in Young People
Source: Front Psychiatry. 2019 Nov 13;10:759. doi: 10.3389/fpsyt.2019.00759 (PMC6865844; doi:10.3389/fpsyt.2019.00759)
Supplement: Supplementary file 1 [file DataSheet_1.docx]

Appendix - Articles Excluded After Full-Text Screening

| Reference | Reason for Exclusion |
| --- | --- |
| Calear, A.L., Christensen, H., Mackinnon, A., Griffin, K. M., O’Kearney, R. (2009) The YouthMood Project: A cluster randomized controlled trial of an online cognitive behavioural program with adolescents. *Journal of Consulting and Clinical Psychology* 77(6) | Duplicate |
| Cabot, S., Wilkinson, B. (2016) Using mobile-based games as a means for the self-treatment of depression and anxiety in youth. *Serious Games, JCSG* 9894 | Full text not available |
| Christensen, H., Batterham, P. J., Gosling, J. A., Ritterband, L. M., Griffiths, K. M., Thorndike, F. P., Glozier, N., O’Dea, B., Hickie, I. B., Mackinnon, A. J. (2016) Effectiveness of an online insomnia program (SHUTi) for prevention of depressive episodes (the GoodNight Study): A randomised controlled trial. *Lancet Psychiatry* 3(4) | Wrong age-group studied |
| Vangbert, H. C., Lillevoll, K R., Waterloo, K., Elsemann, M. (2012) Does personality predict depression and use of an internet-based intervention for depression among adolescents? *Depression Res Treat* | Not relevant |
| Cavanagh, K., Strauss, C., Cicconi, Fl, Griffiths, N., Wyper, A., Jones, F. (2013) A randomised controlled trial of a brief online mindfulness-based intervention *Behav Res Ther* 51(9) | Wrong age-group studied |
| Mewton, L., Andrews, G. (2015) Cognitive behaviour therapy via the internet for depression: A useful strategy to reduce suicidal ideation. *J Affect Disorders* 170 | Wrong age-group studied |
| Nystrom, M. B. T., Stenling, A. Sjostrom, E., Neely, G., Lindner, P., Hassmen, P., Andersson, G., Martell, C., Carlbring, P. (2017) Behavioral activation versus physical activity via the internet: A randomized controlled trial. *J Affect Disorders* 215 | Wrong age-group studied |
| O’Kearney, R., Kang, K., Gibson, M., Christensen, H., Griffiths, K. (2007) A CBT internet program for depression in adolescents (MoodGYM): Effects on depressive symptoms, attributional style, self-esteem and beliefs about depression. *Innovations and Advances in Cognitive Behaviour Therapy* | Duplicate |
| Van der Zanden, R., Curie, K., Van Londen, M., Kramer, J., Steen, G., Cuikpers, P. (2014) Web-based depression treatment: Associations of client’s word use with adherence and outcome | Not relevant |
| Reid, S. C, Kauer, S. D., Hearps, S. J., Crooke, A. H., Khor, A. S., Sanci, L. A., Patton, G. C. (2011) A mobile phone application for the assessment and management of youth mental health problems in primary care: A randomised controlled trial. *BMC Fam Pract* 12 | Duplicate |
| Merry, S. M. D., Stasiak, K. P. 21.4 SPARX: National implementation of an eMental health service. *J or the American Academy of Child and Adolesc Psych.* 55(10) | Not relevant |
| Carli, V. (2016) Prevention suicidality through online tools: The SUPREME Projects | Duplicate |
| Ren, Z., Li, X., Zhao, L., Yu, X., Li, Z., Lau, L., Ruan, Y., Jiang, G. (2016) Effectiveness and mechanism of internet-based self-help intervention for depression: The Chinese version of MoodGYM. *Acta Psychologica Sina* 48(7) | Full text not available |
| Zwaanswijk, M. Kosters, M. P. (2015) Children’s and parents’ evaluations of FRIENDS for Life: An indicated school-based prevention program for children with symptoms of anxiety and depression | Not relevant |
| Sehlen, S., Ott, M., Marten-Mittag, B., Haimerl, W., Dinkel, A., Duehmke, E., Klein, C., Schaefer, C., Herschbach, P. (2012) Feasibility and acceptance of computer-based assessment for the identification of psychosocially distressed patients in routine clinical care. *Psychother Psychocom Med Psychol* 62(7) | Not relevant |
| Wagner, B., and Maercker, A. (2007) A 1.5-year follow-up of an internet-based intervention for complicated grief. *J Trauma Stress* 20(4) | Wrong age-group studied |
| Darcy, A. M., Dooley, B. (2007) A clinical profile of participants in an online support group. *European Eating Disorders Review* 15(3) | Wrong age-group studied |
| Meiser, B. ,Peate, M., Levitan, C., Mitchell, P. B., Trevena, L., Barlow-Stewart, K., Dobbins, T., Christensen, H., Sherman, K. A., Dunlop, K., Schofield, P. R. (2017) A psycho-educational intervention for people with a family history of depression: Pilot results. *J of Genetic Counseling* 26(2) | Wrong age-group studied |
| Twomey, C., O’Reilly, G., Byrne, M., Bury, M., White, A., Kissane, S., McMahon, A. Clancy, N. (2014) A randomized controlled trial of the computerized CBT programme, MoodGYM for public mental health service users waiting for interventions *Br J Clin Psych* 53(4) | Wrong age-group studied |
| Becker, D. (2016) Acceptance of mobile mental health treatments. *7^th^ International Conference on Emerging Ubiquitous Systems and Pervasive Networks (EUSPN 2016)/The 6^th^ International Conference on Current and Future Trends of Information and Communication Technologies in Healthcare (ICTH-2016)* | Full text not available |
| Hedman, E., Andersson, E., Ljotsson, B., Andersson, G., Andersson, E., Schalling, M., Lindefors, N., Ruck, C. (2012) Clinical and genetic outcome determinants of internet- and group-based cognitive behaviour therapy for social anxiety disorder. *Acta Psychiatrica Scandinavica* 126(2) | Full text not available |
| De Graaf, L. E., Gerhards, S. A., Arntz, A., Riper, H., Metsemakers, J. F., Evers, S. M., Severens, J. L., Widdershoven, G., Huibers, M. J. (2009) Clinical effectiveness of online computerised cognitive-behavioural therapy without support for depression in primary care: A randomised controlled trial *Br J Psych* 195(1) | Wrong age-grup studied |
| Dagoo, J., Asplund, R. P., Bsenko, H. A., Hjerling, S., Holmberg, A., Westh, S., Oberg, L., Ljotsson, B., Carlbring, P., Furmark, T., Andersson, G. (2014) Cognitive behavior therapy versus interpersonal psychotherapy for social anxiety disorder delivered via smartphone and computer: a randomized controlled trial. *J Anx Disorders* 28(4) | Wrong age-group studied |
| S. Lokman, S. S. Leone, M. Sommers-Spijkerman, A. van der Poel, F. Smit and B. Boon (2017) Complaint-Directed Mini-Interventions for Depressive Complaints: A Randomized Controlled Trial of Unguided Web-Based Self-Help Interventions  *J Med Internet Res* 12(5) | Wrong age-group studied |
| R. R. Morris, S. M. Schueller and R. W. Picard (2015)  Efficacy of a Web-based, crowdsourced peer-to-peer cognitive reappraisal platform for depression: Randomized controlled trial *JMIR* 17(3) | Wrong age-group studied |
| N. Younes, A. Chollet, E. Menard and M. Melchior (2015)  E-mental health care among young adults and help-seeking behaviors: a transversal study in a community sample. *JMIR* 17(5) | Wrong age-group studied |
| Frazier, P., Meredith, L., Greer, C., Paulsen, J. A., Howard, K., Dietz, L. R., Qin, K. (2015) Randomized controlled trial evaluating the effectiveness of a web-based stress management program among community college students. *Anxiety Stress Coping* 28(5) | Wrong age-group studied |
